# The Transcriptomic Response Along an Urbanization Gradient Is Also Temperature‐Dependent in a Freshwater Insect

**DOI:** 10.1111/mec.70511

**Published:** 2026-08-17

**Authors:** Katarzyna Dudek, Guillaume Wos

**Affiliations:** ^1^ Institute of Environmental Sciences Jagiellonian University Kraków Poland; ^2^ Institute of Nature Conservation Polish Academy of Sciences Kraków Poland

**Keywords:** Climate change, gene expression, growth chambers experiment, impervious surface, *Ischnura elegans*, WGCNA

## Abstract

Urbanization has become one of the leading causes of habitat transformation affecting ecosystem functioning. However, the transcriptomic response to urbanization remains poorly understood. Here, we conducted a transcriptomic analysis along an urbanization gradient under different warming temperatures. We further explored the genes underlying phenotypic variation along an urbanization gradient using weighted gene co‐expression network analysis (WGCNA). We collected adult females in eight ponds spanning an urbanization gradient (percentage of impervious surface) in Southern Poland and raised their larvae in growth chambers under three temperature treatments: current 22°C, + 3°C, and + 6°C above current temperature. When larvae reached the prefinal instar, they were phenotyped for traits related to development and size, and collected for a gene expression analysis. We found that 24 genes varied in expression along a continuous urbanization gradient and were related to proteolysis, extracellular organization and perception. In addition, some genes showed a significant interaction urbanization × temperature and were associated with detection of chemical stimulus and perception. The WGCNA revealed two gene modules associated with urbanization and some phenotype traits, especially with development time. Genes associated with phenotypic differentiation along the urbanization gradient were part of many different pathways including signal transmission, cell cycle and DNA repair. In conclusion, we demonstrated that the transcriptomic response along an urbanization gradient was context‐dependent, here on temperature, suggesting that some genes responding to urbanization also exhibited thermal plasticity. Our work highlights the importance of urbanization in inducing phenotypic and gene expression changes in organisms.

## 1 Introduction

Urbanization has become one of the leading causes of habitat transformation affecting ecosystem functioning. Urbanization is rapidly expanding, threatening terrestrial biodiversity (van Vliet 2019) by altering the abiotic and biotic environment (Grimm et al. 2008). One of the main consequences of urbanization is a rise in temperatures and the formation of the ‘urban heat island’ due to the high density of concrete structures absorbing heat (Grimm et al. 2008; Tam et al. 2015). Consequently, cities warm up faster compared to rural habitats, and urban populations may experience different temperature regimes. Hence, along an urbanization gradient, organisms often show variable life‐history strategies and phenotypic differentiation, with urban populations being generally smaller and developing faster (Brans et al. 2018; Palomar et al. 2023; Tüzün et al. 2017). However, the genetic basis of urban adaptation remains poorly understood but has gained interest over the past few years (Babik et al. 2023; Winchell et al. 2023).

Urbanization has emerged as a strong and widespread source of selection and an important driver of evolutionary changes affecting both plant and animal communities (Alberti et al. 2017). For example, previous studies in insects reported effects of urbanization on size, with a reduction in body size in urban compared with rural populations in bumblebee species (Eggenberger et al. 2019), or the opposite pattern in moth species (Merckx et al. 2018). Similar observations were made in other organisms, with larger flowers in urban populations of the white clover *Trifolium repens* (Santangelo et al. 2020) or a reduced body mass and size in urban areas in the house sparrow *Passer domesticus* (Meillère et al. 2015). As these differences typically persist in common garden experiments, these studies have provided strong support for a genetic basis of urban adaptation. However, insights into the genetic basis underlying this phenotypic differentiation between urban and rural populations remain limited (Mueller et al. 2013). To date, genetic analyses based on SNP genotyping have provided evidence of key metabolic pathways important in urban adaptation. For instance, genome scan analyses revealed that genes involved in the immune system in the mouse *Peromyscus leucopus* (Harris et al. 2013), in synapse organization in the damselfly *Ischnura elegans* (Babik et al. 2023) or in genes related to development and morphology in the lizard *Anolis cristatellus* (Winchell et al. 2023) were under selection in an urban environment. However, only a few studies have explored the transcriptomic basis along an urbanization gradient, which may provide new insights into functional genes showing constitutive expression differences, for example, related to the immune system or oxidative stress in the great tit *Parus major* (Watson et al. 2017).

We proposed, here, to explore the transcriptomic response to urbanization by conducting a genome‐wide transcriptomic analysis to track changes in gene expression along a continuous urbanization gradient, measured here as the percentage of impervious surface. We further linked gene expression with phenotypic data using weighted gene co‐expression network analysis (WGCNA; Langfelder and Horvath 2008) to identify relevant sets of genes underlying phenotypic differentiation along an urbanization gradient.

Moreover, in the context of urbanization, it is generally expected that urban populations cope differently with a rise in temperature compared to rural populations, as urban areas are characterized by higher temperatures, that is, ‘urban heat islands’, or a higher frequency of extreme events (Angilletta et al. 2007). For instance, rural populations showed lower survival compared to urban ones in response to a rise in temperature in the damselfly *Coenagrion puella* (Tüzün et al. 2017; Tüzün and Stoks 2021). It was also demonstrated that there were differences in mass and growth rate between urban and rural populations at 20°C but not at 24°C in the damselfly *Ischnura elegans* (Palomar et al. 2023) or that both urbanization and temperature affected butterfly phenology (Diamond et al. 2014) and thermal tolerance in the water flea *Daphnia magna* (Brans et al. 2017). Hence, differentiation in life‐history traits along an urbanization gradient may also be dependent on temperature. To go one step further, we tested to what extent the response along an urbanization gradient was modulated by temperature at both the phenotypic and transcriptomic level, which is important in the current context of global warming.

**FIGURE 1:**
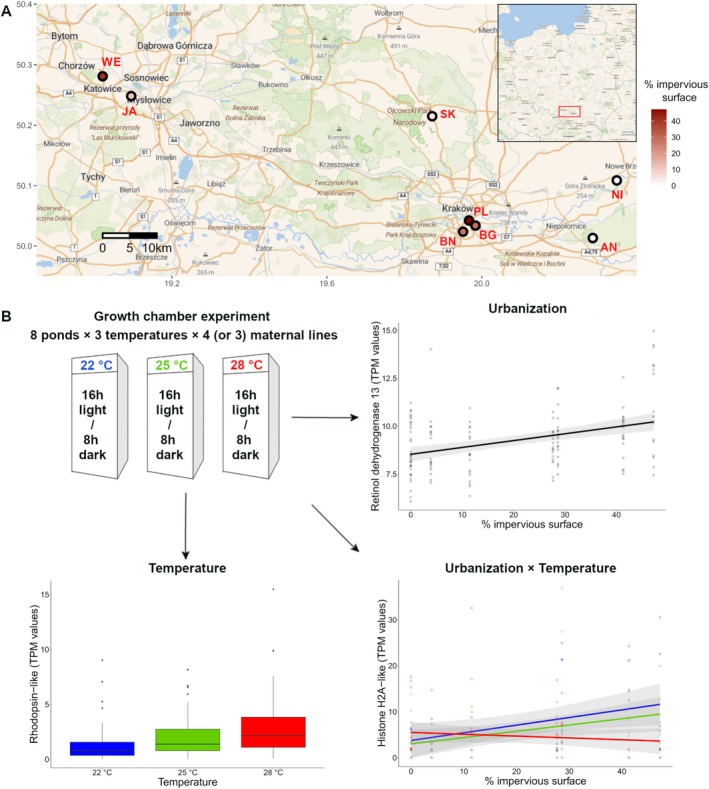
(A) Location of the eight ponds sampled for adult females coloured by the percentage of impervious surface (proxy of urbanization). Abbreviations can be found in Table S1. (B) Overall design of the experiment. Larvae from field‐collected females were raised in growth chambers under three temperature treatments: 22°C, 25°C and 28°C until F‐1 larval stage and collected for gene expression analysis. The plots show examples of genes significantly affected by urbanization, temperature and urbanization × temperature. Grey areas represent standard error.

In the present study, we conducted a common garden experiment on the damselfly *I. elegans* to explore variation in the transcriptome along an urbanization gradient. We raised larvae in growth chambers from field‐collected females from eight ponds located along an urbanization gradient in southern Poland (Figure 1A). Larvae were raised under one of three thermal regimes: current temperature 22°C, + 3°C, and +6°C above current temperature, corresponding to different climate change scenarios modelled by the Intergovernmental Panel on Climate Change (Masson‐Delmotte et al. 2021). At the end of the experiment, larvae were phenotyped for traits related to size and development and collected for gene expression analysis. First, we specifically tested for the effect of urbanization, temperature and their interaction on gene expression. We expected temperature to be the main driver of gene expression changes because of its importance in the development of ectotherms. The effect of urbanization and of the interaction urbanization × temperature are expected to affect a limited number of genes, as in our system, geographic distances are relatively short between ponds and gene flow is relatively high. Next, we correlated gene expression data with phenotypic traits using WGCNA to identify groups of correlated genes underlying phenotypic variation along an urbanization gradient.

## 2 Methods

### 2.1 Study System and Sampling

Our study system is the damselfly *I. elegans* , a common species in Europe (Dijkstra and Schröter 2020). At central latitudes, including Poland, populations are generally uni‐ and bivoltine, that is, one or two generations per year (Corbet et al. 2006). At the same latitude, populations are not genetically isolated; it was previously demonstrated that there is no difference in the genetic structure between urban and rural ponds (including some ponds sampled in the present study) in *I. elegans* suggesting relatively high gene flow at a local scale (Babik et al. 2023).

We sampled eight ponds located along an urbanization gradient in southern Poland (Figure 1A). We quantified the level of urbanization based on the percentage of impervious surface, a common measure used to assess the effects of urbanization (Morse et al. 2003), from the high‐resolution layer database (20 m resolution) (EEA 2020) using Quantum‐GIS (QGIS Development Team 2017). For this, we created a circular buffer of 1 km around each sampling site and calculated the percentage of impervious surface in each buffer (Sniegula and Wos 2026). Across the eight ponds, the percentage of impervious surface ranges from 0% to 47.21% (Table S1). Additional information on the eight ponds is provided in Figure S1.

We collected copulating females in each pond between June 16, 2025 and June 22, 2025 to ensure minimum temporal variation so that the collected females belonged to the same cohort. In each pond, we collected between four and nine females with a sweeping net and each female was placed in an individual plastic jar with wet filter paper. Then, plastic jars with females (maternal lines hereafter) were brought back to the laboratory and left at room temperature and under natural photoperiod for egg laying, which typically occurs within 3 days. After egg laying, each egg clutch was placed in an individual container filled with 300 mL of dechlorinated tap water and transferred into a growth chamber at 22°C, LD 16 h/8 h.

### 2.2 Growth Chamber Experiment

Eggs hatched between July 5, 2026 and July 11, 2026. For the growth chamber experiment, we randomly selected 4 maternal lines per pond, except for one pond, Janow, where only three females laid eggs, totaling 8 ponds × 4 (or 3) maternal lines = 31 maternal lines. After hatching, for each maternal line separately, we prepared seven plastic cups filled with 100 mL of dechlorinated tap water, each cup containing 10 larvae randomly selected (31 maternal lines × 7 cups × 10 larvae). Larvae were raised in these group cups for 10 days at 22°C, LD 16 h/8 h to increase survival. During these 10 days, larvae were fed twice a day (week days) and once a day (weekend days) with laboratory‐cultured *Artemia nauplii*.

After 10 days, larvae were split into individual cups and transferred into different growth chambers corresponding to the three temperature treatments: constant 22°C, constant 25°C and constant 28°C and under the same photoperiod of LD 16 h/8 h. To avoid an abrupt change in temperature for larvae exposed to 28°C, they were first transferred for 3 days to 25°C and then to 28°C. The three temperatures: current 22°C, +3°C, and +6°C above the current temperature correspond to different climate change scenarios modelled by the Intergovernmental Panel on Climate Change (Masson‐Delmotte et al. 2021). These temperatures are not stressful, as the weekly average temperature can reach 25°C in some ponds in the middle of summer and 28°C occurs during hot days (Figure S1). In total, we started the experiment with 8 ponds × 3 temperatures × 4 (or 3) maternal lines × between 12 and 24 individuals = 1275 larvae. We did not use the same number of larvae for each pond and treatment. First, to compensate for the fact that in one pond: Janow, we had only three maternal lines. Therefore, we raised more individuals per maternal line for this pond. Secondly, we also increased the sample size in the 22°C treatment to ensure we had enough individuals reaching the specific development time for the gene expression analysis and further analyses, as in this treatment, growth and development may vary greatly. Larvae were raised under these conditions for 90 days corresponding to the duration of the summer period in nature. For the experiment, six incubators were used (two incubators per temperature), trays holding individual cups were rotated between incubators every week, and trays were also rotated within an incubator twice per week. Larvae were fed twice a day (week days) and once a day (weekend days) with laboratory‐cultured *Artemia nauplii*. During this 90‐day period, larvae were checked daily for their development stage. When larvae reached the prefinal instar (F‐1 hereafter), in the morning and before feeding, they were phenotyped and two larvae per maternal line and treatment were preserved in RNAlater (ref: R0901; Sigma‐Aldrich) for the gene expression analysis. The other larvae that did not moult to F‐1 before the end of the experiment and were at lower instars were included in the analysis of survival but were not phenotyped.

### 2.3 Life‐History Traits

In total, 989 larvae entered F‐1 (*N* = 342 larvae at 22°C; 305 at 25°C and 342 at 28°C) and were phenotyped for four life‐history traits: development time (number of days between hatching and reaching F‐1), wet mass (mass hereafter), head width and wing pad length. Head width is commonly used as a proxy for body size in odonates (Corbet 1999) and wing pad length is related to wing size and adult dispersal ability. During the 90‐day period, survival was also monitored every week.

### 2.4 Sequencing and Data Processing

For the gene expression analysis, we collected a total of 144 larvae (8 ponds × 3 temperatures × 3 maternal lines × 2 individuals). First, total RNA was extracted from each individual using RNAzolRT according to the manufacturer's instructions, and its purity and quantity were measured on NanoDrop and integrity was assessed by automated agarose electrophoresis on the Agilent TapeStation system. Library preparation and sequencing were performed at Novogene. Sequencing was conducted on NovaSeq X Plus Series (150 pb paired‐end). Sequencing generated between 19.6 and 42.2 million raw read pairs per individual. The data are available from the Sequence Read Archive under accession PRJNA1453760.

For the data processing, raw reads were first trimmed to remove adapter sequences using cutadapt 5.2 (Martin 2011). Next, reads were mapped on the *I. elegans* reference genome using the corresponding annotation from the Darwin Tree of Life Project (project ID: PRJEB46264; The Darwin Tree of Life Project Consortium 2022) using hisat2 2.2.1 (Kim et al. 2019). After the alignment, the number of reads mapped to each gene was counted with featurecounts 2.1.1 (Liao et al. 2019) and only the uniquely mapped reads were retained (between 13.9 and 30.5 million reads per individual). Information on sequencing data and alignment is available in Table S1.

### 2.5 Differential Expression Analysis

Read counts were analysed using glmmSeq (Lewis et al. 2021) on R (R Core Team 2024; RStudio Team 2024), which allows fitting a general linear mixed model including nested random effects and is designed to implement continuous variables. The model used was: glmmSeq(gene expression data ~ urbanization × temperature + (1|Pond/maternal line)). Urbanization was the percentage of impervious surface and was used as a continuous variable, temperature corresponded to the three temperature treatments (22°C, 25°C and 28°C) with 22°C being the reference temperature. Maternal line nested within pond (three maternal lines per pond) was the random factor. For the gene expression analysis, we removed genes with low or no expression values using the function filterByExpr from the edgeR package (Robinson et al. 2010). Among the 19,460 genes annotated in the *I. elegans* genome, 13,322 were retained for the analysis. We followed the procedure described in the glmmSeq package and included in the model gene dispersion estimates (“dispersion” option) and a scaling factor to take into account variation in library size across samples (“SizeFactors” option). *p*‐values were adjusted using the glmmQvals function implemented in the package, genes were considered as differentially expressed if adjusted *p*‐values < 0.05. For the effects of the continuous variable “urbanization”, the direction of the expression changes along the gradient was indicated by the sign of the regression coefficient “‐” for a decrease in expression values as the percentage of impervious surface increases or “+” for an increase in gene expression values. For the effects of temperature, 22°C was the reference, a negative coefficient between 22°C and 25°C (or between 22°C and 28°C) indicates lower expression at 25°C (or 28°C) and a positive coefficient indicates higher expression at 25°C (or 28°C). Based on this, we defined three categories: (1) genes decreasing their expression in response to temperature if coefficients were negative between 22°C and 25°C and between 22°C and 28°C, (2) genes increasing their expression if coefficients were all positive and (3) genes with different directions if the signs were different.

### 2.6 Weighted Gene co‐Expression Network Analysis (WGCNA)

In order to link our phenotypic and gene expression data, we also performed a WGCNA following the general procedure described in the WGCNA package (Langfelder and Horvath 2008). The WGCNA is a method used to correlate gene expression data with other variables such as phenotypic or environmental data. We ran a WGCNA to find clusters of highly correlated genes associated with our phenotypic data, urbanization and temperature. As input files, we included gene expression data; genes with low or no expression values were removed. The phenotypic data included development time, head width, mass and wing pad length measured on the 144 individuals. Temperature and urbanization values measured as the percentage of impervious surface were also added as variables. First, we created a dendrogram to detect outliers among our 144 samples and no outliers were detected (Figure S2). Next, we created the network by first selecting a soft thresholding power. According to the guidelines, the soft thresholding power corresponds to the value where the correlation coefficient is higher than 0.8 and where the mean connectivity value is between 50 and 100, which corresponds to a value of 4 (Figure S3). We constructed a signed gene expression network using the blockwiseModules function with the following parameters: minModuleSize = 50, mergeCutHeight = 0.3 and deepSplit = 2 (Figure S4). Finally, each gene module was correlated with each phenotypic trait, urbanization values and temperature, and correlations were considered significant if FDR‐adjusted *p*‐values < 0.05. In addition, for significant gene modules associated with urbanization, we also described the hub genes defined, here, as the ten genes with the highest eigengene‐based connectivity values (the most representative genes within a module in terms of connections with other genes) and that were annotated in the *I. elegans* genome (‘Gene name’ available). We also ran a module preservation analysis using the original dataset as a reference set and the target set was estimated using permutations of the original dataset (N permutations = 200). We reported the Z summary scores and median rank for each module (Table S2), with Z scores ≤ 2 indicating no module preservation, 2 ≤ Z scores ≤ 10 indicating moderate preservation and Z scores ≥ 10 indicating strong preservation. Median rank lists the different modules from the most preserved to the least preserved. In our dataset, the Z scores are always greater than 10, except for two modules (brown and blue) where the Z scores are just below 10, indicating overall strong module preservation.

### 2.7 Gene Ontology and Enrichment Analysis

The gene expression analysis and WGCNA were both complemented with a gene ontology (GO) term enrichment analysis. For this, we created a custom annotation following the procedure described in Wos et al. (2023). Briefly, we assigned GO terms to each *I. elegans* gene if they had the same name and function and were associated with the same GO terms in at least two other insect species in the Uniprot database (released version 28 January 2026; Uniprot Consortium 2015). In total, we assigned GO terms to 7581 *I. elegans* genes. The GO term enrichment analysis was performed using BiNGO v3.0.5 software (Maere et al. 2005) in Cytoscape v3.9.1 (Shannon et al. 2003) with the most recent gene ontology annotation (released version 23 January 2026; The Gene Ontology Resource; Ashburner et al. 2000). *p*‐values were adjusted for multi‐testing, and GO terms were considered as significantly enriched if adjusted *p*‐values < 0.05. The GO term enrichment analysis was complemented by REVIGO clustering to remove redundant GO terms and group them based on semantic similarities (Supek et al. 2011).

### 2.8 Statistical Analysis

Statistical analyses were performed in R (R Core Team 2024; RStudio Team 2024). We ran principal component analyses on the four phenotypic traits and on the gene expression data for each temperature treatment separately. The phenotypic traits and gene expression data were scaled and centered. Survival was analysed using a generalized linear mixed model (glmmTMB; Magnusson et al. 2017) with a binomial distribution, with the interaction urbanization × temperature as a fixed factor and maternal line nested within pond as a random factor.

## 3 Results

### 3.1 General Effects of Urbanization and Temperature at the Phenotypic and Transcriptomic Level

**FIGURE 2:**
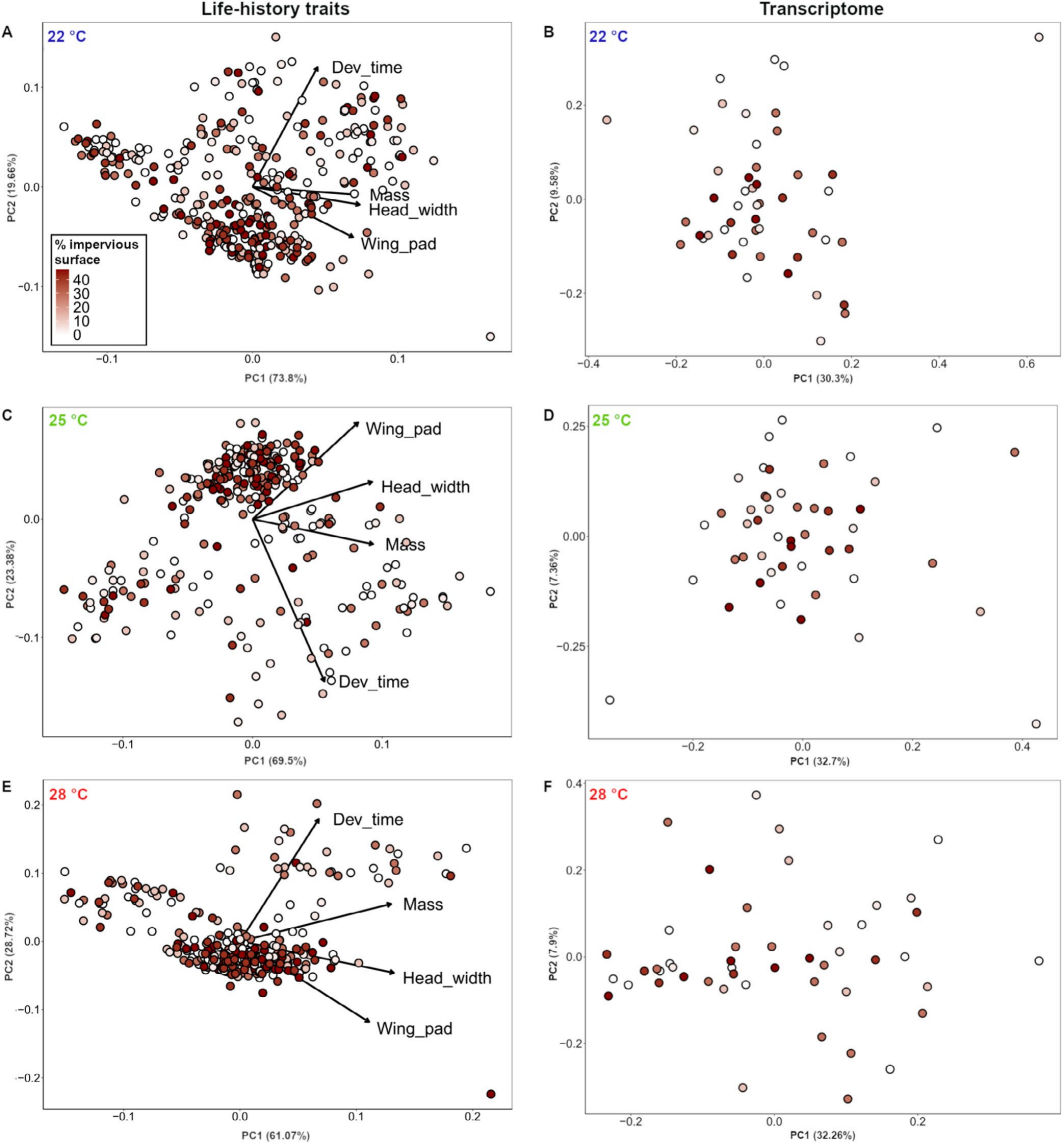
Principal component analysis performed on the life‐history traits (left column) and gene expression data (right column) for each temperature treatment. Each point depicts one individual coloured by the percentage of impervious surface of their pond of origin (proxy of urbanization).

Among the 1275 larvae raised in growth chambers, 178 larvae died over the course of the experiment; urbanization, temperature, and their interaction had no significant effect on mortality. PCA plots at 22°C showed faster development when the percentage of impervious surface increased (PC2 axis; Figure 2A). The other traits separated along the PC1 axis and the link with urbanization was less clear. At the transcriptomic level, there was no differentiation along the urbanization gradient (Figure 2B). At 25°C, we observed similar trends to those observed at 22°C, with a separation along the urbanization gradient along the PC2 axis and no differentiation at the transcriptomic level (Figure 2C,D). At 28°C, almost all individuals, independently of their pond of origin, tended to cluster together; the effects of urbanization were less clear at the phenotypic level and not detectable at the transcriptomic level (Figure 2E,F).

### 3.2 Differential Expression Analysis Along an Urbanization Gradient Under Different Temperatures

Generalized linear model analysis of the transcriptomic data revealed genes significantly affected by urbanization, temperature and their interaction. In total, 24 genes were significantly affected by urbanization (Table S3), of which 15 increased their expression with the percentage of impervious surface (Figure 1B) and nine decreased their expression (Table 1). Gene ontology term enrichment analysis revealed six enriched GO terms related to proteolysis, extracellular structure organization and sensory perception of sweet taste. Among the genes that increased their expression, we found genes involved in hydrolysis and oxidoreduction, lipid process or regulation of transcription (Table 1). Those that decreased their expression are involved in proteolysis, sensory perception or lipid catabolic process.

**TABLE 1:** Lists of genes significantly affected by urbanization (percentage of impervious surface) and by the interaction urbanization × temperature. For the effects of urbanization, the column ‘Coefficient’ indicates the regression coefficient: Negative values indicate negative correlations between gene expression and the percentage of impervious surface and positive values indicate a positive correlation. The last column shows the main functions of each gene. Only the genes described in the annotation are shown.

Variable	Gene ID	Gene name	Coefficient	Functions
Urbanization	LOC124169412	Putative gustatory receptor	−0.05458	Sensory perception of taste
	LOC124167117	A disintegrin and metalloproteinase with thrombospondin motifs 9‐like	−0.03554	Proteolysis, zinc ion binding
	LOC124166803	A disintegrin and metalloproteinase with thrombospondin motifs	−0.03434	Proteolysis, binding to a metal ion
	LOC124164316	Adhesion G protein‐coupled receptor L4‐like	−0.03408	G protein‐coupled receptor activity
	LOC124167088	A disintegrin and metalloproteinase with thrombospondin motifs 9	−0.02221	Proteolysis, zinc ion binding
	LOC124163768	Phospholipase A1‐like	−0.01671	Lipid catabolic process
	LOC124167541	Retinol dehydrogenase 13‐like	0.004191	Oxidoreductase activity
	LOC124170374	Zinc finger protein 35‐like	0.015281	RNA polymerase II transcription regulatory region
	LOC124171115	Histone H2A‐like	0.026702	Protein heterodimerization activity
	LOC124158165	Transmembrane protease serine 9‐like	0.038889	Serine‐type endopeptidase activity, proteolysis
	LOC124162240	4‐coumarate‐CoA_ligase CCL1‐like	0.040173	Long‐chain fatty acid‐CoA ligase activity
	LOC124158718	Putative nuclease HARBI1	0.050735	Hydrolase activity, nuclease activity
	LOC124167937	Putative nuclease HARBI1	0.064307	Hydrolase activity, nuclease activity
Urbanization × temperature	LOC124169412	Putative gustatory receptor	/	Sensory perception of taste
	LOC124171115	Histone H2A‐like	/	Protein heterodimerization activity
	LOC124164970	Myb/SANT‐like DNA‐binding domain‐containing protein 3	/	DNA binding
	LOC124160940	Probable chitinase 10	/	Chitinase activity
	LOC124171070	Putative nuclease_HARBI1	/	Hydrolase activity, nuclease activity
	LOC124169104	Lysine‐specific demethylase 6B‐like	/	Methylation, regulation gene expression
	LOC124164438	Myb/SANT‐like DNA‐binding domain‐containing protein 3	/	DNA binding
	LOC124164497	Protein ALP1‐like	/	Hydrolase activity, nuclease activity
	LOC124172254	Myb/SANT‐like DNA‐binding domain‐containing protein 3	/	DNA binding

In total 6311 genes were affected by temperature; of those, 4381 genes went in the same direction and decreased their expression values at both 25°C and 28°C compared with 22°C (Table S3; Figure S5). The GO term enrichment analysis revealed 63 enriched GO terms; after removing redundant GO terms, 24 remained and were grouped into six categories by REVIGO: RNA processing, metabolic process, cellular process, ribonucleoprotein complex biogenesis, cellular response to stress and cellular localization. RNA processing is the largest category and included genes related to various metabolic and catabolic processes of macromolecules. Followed by cellular localization containing genes related to transport and protein localization. Cellular response to stress included many genes related to DNA repair. Among the 6311 genes, 794 went in the same direction and increased their expression values at both 25°C and 28°C compared with 22°C with only one enriched GO term related to carbohydrate metabolic process (Figure 1B). We also performed a functional categorization and grouped genes based on their GO terms: metabolic and primary metabolic process, response to stimulus, localization and transport. Finally, 1136 genes went in different directions at 25°C and 28°C compared with 22°C (Figure S5); no GO terms were significantly enriched. Functional categorization showed genes involved in metabolic process, regulation of biological process and regulation of transcription.

We found 25 genes showing a significant interaction urbanization × temperature with nine genes described (Figure 1B; Table 1). Six GO terms were significantly enriched and associated with the detection of chemical stimulus and sensory perception of taste. The six enriched GO terms included only one gene: *PUTATIVE GUSTATORY RECEPTOR* (LOC124169412), which is involved in sensory perception (Table S3). The other genes affected by the interaction are involved in methylation, DNA binding, hydrolysis and chitinase activity (Table 1). Among those, three genes, *PROBABLE CHITINASE 10* (LOC124160940), *PUTATIVE GUSTATORY RECEPTOR* (LOC124169412) and one of the *myb/SANT‐like DNA‐BINDING DOMAIN‐CONTAINING PROTEIN 3* (LOC124164438) showed a decrease in expression values along the urbanization gradient at 22°C and a consistent increase in expression at both 25°C and 28°C (Figure S6). For three genes: *HISTONE H2‐LIKE* (LOC124171115), *LYSINE‐SPECIFIC DEMETHYLASE 6B‐LIKE* (LOC124169104) and one *myb/SANT‐like DNA‐BINDING DOMAIN‐CONTAINING PROTEIN 3* (LOC124172254), the expression values went in the same direction at 22°C and 25°C and in a different direction at 28°C (Figure 1B; Figure S6). For the other genes, the patterns were more complex (Figure S6).

### 3.3 Correlation Between Gene Expression, Phenotype and Urbanization

**FIGURE 3:**
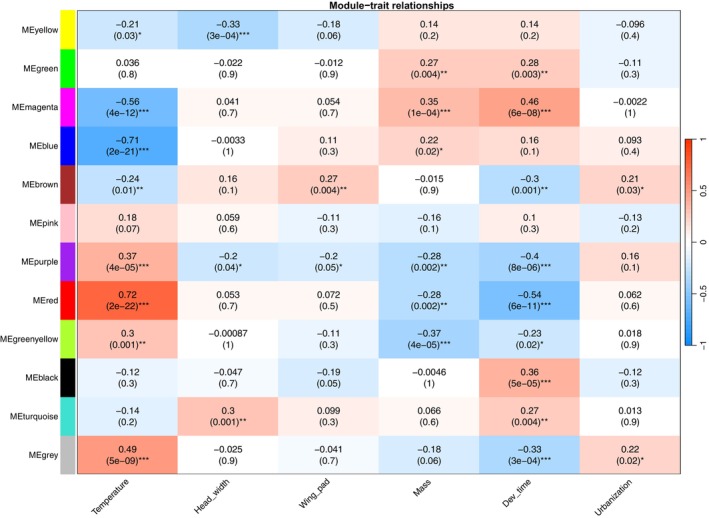
Results of the weighted gene co‐expression network analysis showing correlation coefficients and FDR‐adjusted *p*‐values (in parentheses) between the life‐history traits, the percentage of impervious surface (urbanization) and temperature, and each gene module, depicted by different colours on the left, corresponding to groups of correlated genes. Significance is indicated by *p < 0.05, **p < 0.01 and ***p < 0.001.

The WCGNA returned 12 gene modules, two of those were significantly associated with urbanization and also with some phenotypic traits and temperature (Figure 3). The brown module was positively associated with urbanization and wing pad length and negatively with developmental time and temperature. The module included 1484 genes and the GO term enrichment analysis revealed 80 enriched GO terms. After removing redundant GO terms, 18 enriched GO terms remained grouped into eight clusters: DNA repair, microtubule‐based process, cell cycle, cell cycle process, response to stimulus, chromosome organization, cell division and regulation of the cell cycle (Figure S7). DNA repair was the largest cluster with the highest number of genes and also contained genes related to DNA amplification, primary metabolism and response to stress. The hub genes of the brown module were involved in cellular organization, cell division and development (Table 2).

**TABLE 2:** Description of the hub genes of the brown and grey modules. Hub genes were defined as the ten genes with the highest eigengene‐based connectivity values (the most representative genes within a module in terms of connections with other genes) and that were annotated in the *I. elegans* genome (‘Gene name’ available).

Gene ID	kME	Gene name	Functions
*Gene module: brown*			
LOC124155616	0.92	Protein DEK	Chromatin‐organization
LOC124156386	0.90	Mitotic checkpoint protein BUB3	Cell division
LOC124153451	0.90	Aurora kinase C‐like	/
LOC124154228	0.89	Microtubule‐associated protein futsch‐like	Axogenesis
LOC124156428	0.89	Citron rho‐interacting kinase	Actomyosin structure organization
LOC124155762	0.89	Cell division control protein 45 homologue	Cell division
LOC124153947	0.89	Condensin complex subunit 2‐like	/
LOC124153908	0.88	Condensin complex subunit 3	Cell division
LOC124157398	0.88	Serine‐rich adhesin for platelets‐like	Regulation transcription/morphogenesis
LOC124156436	0.86	Chromatin assembly factor 1 p55 subunit	Chromatin organization
*Gene module: grey*			
LOC124173130	0.61	Zinc finger and SCAN domain‐containing protein 2‐like	Regulation of transcription
LOC124170162	0.60	Zinc finger protein 260‐like	Epigenetic regulation of gene expression
LOC124173072	0.59	Oocyte zinc finger protein XlCOF6‐like	Regulation of transcription
LOC124173216	0.59	Zinc finger protein 184‐like	Regulation gene expression
LOC124173045	0.57	Oocyte zinc finger protein XlCOF6‐like	Regulation of transcription
LOC124170382	0.56	Zinc finger protein 271‐like	Negative regulation of cell differentiation
LOC124173294	0.55	Zinc finger protein 62‐like	Regulation of transcription
LOC124170161	0.54	Oocyte zinc finger protein XlCOF6‐like	Regulation of transcription
LOC124172537	0.54	Zinc finger protein 37 homologue	Regulation of transcription
LOC124173081	0.54	Zinc finger protein 2 homologue	Regulation of transcription

The grey module was positively associated with urbanization and temperature, and negatively associated with developmental time. The module included 5012 genes with 29 enriched GO terms. After removing redundant GO terms, 11 remained grouped into five clusters: proteolysis, cell surface receptor signalling pathway, modulation of process of another organism, monoatomic ion transmembrane transport and cilium organization (Figure S8). The cluster ‘cilium organization’ also contained GO terms related to epigenetic regulation of gene expression. The cluster ‘cell surface receptor signalling pathway’ contained GO terms regulation of gene silencing. The hub genes of the grey module were all part of the zinc finger protein family and regulate transcription also through epigenetic mechanisms (Table 2).

## 4 Discussion

We aimed at exploring the transcriptomic response along an urbanization gradient in the context of global warming in a freshwater insect. We demonstrated that some genes related to proteolysis, cellular organization and perception had constitutive expression changes along the urbanization gradient, measured as the percentage of impervious surface. Moreover, we also found that along the urbanization gradient, the expression of some genes was also dependent on temperature. Our results showed that the phenotypic differentiation along the urbanization gradient in traits related to body size and development also had a transcriptomic basis involving metabolic pathways associated with development and regulation of transcription.

It has often been assumed that urbanization acts as a barrier and contributes to the genetic isolation of populations by reducing gene flow and increasing genetic drift even over short geographic distances (Miles et al. 2019). This is more likely to be the case in organisms with limited dispersal ability. In our case, urbanization does not seem to prevent individual movement, as no genetic structure was found between ponds (including some of our sampled ponds) in Southern Poland (Babik et al. 2023). In addition, in damselflies and dragonflies, a high degree of kinship between populations separated by less than 150 km (Watts et al. 2010) and migration distances of up to 88 km per year (Flenner and Sahlén 2008) were demonstrated. In *I. elegans* , previous studies showed high rates of migration between ponds located less than 1 km from each other (Conrad et al. 1999) and that females tended to migrate farther than males in rural habitats (Gall et al. 2017). Consequently, we may expect high migration rates in our study system, which may explain the relatively small number of genes differentially expressed. In this context, available theories suggest that high levels of gene flow tend to favour genomic homogenization between populations, consequently, only a small set of genes with large phenotypic effects should differ (Yeaman and Whitlock 2011). Our results tended to support the theoretical predictions, as no differentiation along the urbanization gradient was observed at the whole‐transcriptomic level, and only 24 genes were significantly affected by urbanization and involved in major biological functions or regulatory processes, but the importance of these genes on the phenotype remains to be determined. Previous transcriptomic analyses in birds also found a relatively small number of genes differentially expressed between urban and rural populations that were involved in the immune system in *P. major* (Watson et al. 2017) or in multiple different pathways, that is, stress, energy or lipid metabolism, in *Falco tinnunculus* (Damiani et al. 2024). In insects, a previous transcriptomic study found no impact of urbanization on gene expression between urban and rural populations separated by 50 km in the ant *Temnothorax nylanderi* (Smith et al. 2025). Previous genome scan analyses between urban and rural populations revealed genes related to synapse organization and sensory perception in *I. elegans* (Babik et al. 2023), to signal transduction and metabolic process in the bumblebee *Bombus lapidarius* (Theodorou et al. 2018), to skeletomuscular development and morphology in the lizard *Anolis cristatellus* (Winchell et al. 2023) or to catalysis and mRNA processing in the acorn ant *Temnothorax nylanderi* (Khimoun et al. 2020). Unlike previous transcriptomic studies, our results did not reveal constitutive expression differences in stress‐responsive genes but instead in genes involved in proteolysis, development, perception of the environment, regulation of transcription or lipid process. We identified some genes belonging to pathways that overlap with previous genome scan studies for example, regulation of transcription, sensory perception or development, revealing some important adjustments in development and cognition/behaviour along an urbanization gradient and might be important for urban adaptation. But this would require further analyses to identify signatures of urban adaptation in these genes, because we cannot exclude other evolutionary forces such as maternal effects, plasticity, epigenetics or genetic drift that can trigger differential gene expression that may be adaptive or not. In particular, maternal effects play an important role in insects' evolution and adaptation and contribute to developmental differences even within populations (Dingle 1991). In our study, we collected only three maternal lines per pond for the transcriptomic analysis, which may not fully capture the diversity of the response observed in the field. These results highlight, on the one hand, key regulatory pathways associated with urbanization that may be shared across organisms and, on the other hand, that the response to urbanization also involves several genes/metabolic pathways that may be species‐ or context‐specific. Indeed, along urbanization gradients, the environment changes in a complex and multivariate manner. We may hypothesize that selection is more likely to target a couple of genes in multiple metabolic pathways rather than genes in one specific pathway.

As predicted, temperature was the most important driver of gene expression differences, as it is an important factor governing the development and physiology of ectotherms (Angilletta Jr. et al. 2004), which may explain the high number of genes differentially expressed. In aquatic insects, water temperature plays a fundamental role in development, growth rate, body size at maturity/emergence and behaviour (Ward and Stanford 1982). Moreover, the effects of temperature in *I. elegans* are more important on larval development under long days than short days, which is the case in our study (LD 16 h/8 h) (Norling 2021). As temperature increases, trade‐offs may be expected between faster development and other functions such as the immune system in *Drosophila* (González‐Rete et al. 2019). Most of the genes identified consistently decrease their expression at both 25°C and 28°C compared with 22°C suggesting potential trade‐offs between development and other functions related to the stress response and DNA repair mechanisms. The overrepresentation of GO terms related to catabolic processes of macromolecules and proteins and transport may be due to the fact that a rise in temperature affects metabolism and protein turnover (González‐Tokman et al. 2020) and was supported by previous studies in damselflies (Swaegers et al. 2020; Wos et al. 2023). Moreover, warming temperatures also affect carbohydrate metabolism and energy balance (González‐Tokman et al. 2020) as demonstrated in the beetle *Tenebrio molitor* (Rho and Lee 2017) and the damselfly *Lestes sponsa* (Wos et al. 2026), which is consistent with our findings, as carbohydrate metabolism was the only enriched GO term among the genes that consistently increased in expression at both 25°C and 28°C. Finally, a lot of genes went in different directions at 25°C and 28°C compared with 22°C and were involved in various general functions without any enriched GO terms. The response to a rise in temperature is not necessarily uniform; some genes may have temperature‐specific expression profiles and are probably expressed or repressed at a certain threshold, as observed, for example, for some heat shock proteins (Fangue et al. 2006).

We also identified genes showing significant interaction between urbanization and temperature. We found only one overrepresented GO term with one gene, *PUTATIVE GUSTATORY RECEPTOR*, involved in sensory perception of taste and chemoreception, which is fundamental for discriminating between substances (Engsontia et al. 2014). Other genes are part of various pathways. For instance, two genes were related to the regulation of expression through epigenetic mechanisms, which allow rapid adjustments to environmental changes in insects (Gupta and Nair 2025). One gene, *PROBABLE CHITINASE 10*, is involved in defence and immunity but also in moulting and development (Arakane and Muthukrishnan 2010). The other genes involved in DNA binding or hydrolysis are ubiquitous; the link with urbanization and temperature is less clear. Overall, our results highlight the context‐dependency of the effects of urbanization even at the transcriptomic level suggesting that some genes associated with urbanization also exhibited some degrees of thermal plasticity.

### 4.1 Transcriptomic Basis of Phenotypic Variation Along an Urbanization Gradient

Along the urbanization gradient, based on the PCA, we found overall faster development, especially at 22°C and 25°C, which was consistent with previous studies showing faster development in *Daphnia magna* (Brans et al. 2018) or faster growth in *I. elegans* (Palomar et al. 2023) in urban areas. This was also supported by the WGCNA as in each of the two significant associations detected, gene modules correlated positively with urbanization and negatively with development time. Temperature was also involved in each association, yet in different directions but confirmed that the effects of urbanization on the phenotype were also dependent on rearing temperature.

The brown module was positively associated with urbanization and wing pad length and negatively with developmental time and temperature. We had an overrepresentation of genes in DNA repair, cell division or response to stimulus, which may reflect some types of cellular stress associated with urbanization and/or temperature, for example, oxidative stress (Isaksson 2015), and the maintenance of genomic integrity. In line, previous studies demonstrated that stressful urban conditions may cause DNA damage in mice (Yauk et al. 2008) and in ants (Konorov et al. 2017). We may assume some metabolic costs associated with DNA repair mechanisms and the maintenance of genome integrity, which may manifest in the phenotype.

The grey module was positively associated with urbanization and temperature, and negatively with development time. The genes underlying the phenotypic differentiation participated in the transmission of information in response to external stimuli, the initiation of molecular cascades, and the regulation of transcription. The grey module included genes involved in information gathering and processing, which are part of pathways by which an organism responds to environmental changes (Kelley et al. 2018). In addition, we also found some genes associated with epigenetic mechanisms. Environmental stress caused by an urban environment may increase epigenetic mutations (Thompson et al. 2022), for example, hypermethylation in Darwin finches (McNew et al. 2017), opening avenues to further explore the role of epigenetics in urban adaptation and in phenotypic plasticity.

Taken together, our results show that urbanization, depicted here by the percentage of impervious surface, induces phenotypic and transcriptomic changes among populations and over relatively short geographical distances. However, our design involves eight ponds distributed along an urbanization gradient without replicate ponds for each level of urbanization, which may limit inference to a broader scale or to other ecological systems. Our common garden setting revealed that the response to urbanization had a genetic basis despite the homogenizing effect of gene flow. Moreover, current climate change, reflected by the different temperature regimes, could further impact the response to urbanization. Along an urbanization gradient, damselflies adjust the expression of some genes differently in response to a rise in temperature, which could amplify the genetic and possibly phenotypic divergence between ponds. However, the question of whether the genes differentially expressed along the urbanization gradient are adaptive remains to be tested.

## 5 Conclusions

Our study explored the transcriptomic response to urbanization under different temperature regimes in a freshwater insect. We found that (1) the transcriptomic response along an urbanization gradient involved genes related to proteolysis, extracellular organization and sensory perception, (2) the response to urbanization was context‐dependent, here on temperature, and (3) the phenotypic differentiation, mostly in development time, observed along an urbanization gradient was also dependent on temperature and was significantly associated with some gene clusters. Hence, our results showed that both urbanization and temperature had clear effects on the larval transcriptome and phenotype but how this translates into adult traits, particularly in terms of fecundity, would require further examinations.

## Author Contributions

G.W. conceived the ideas and designed the methodology; G.W. performed the experiment; K.D. performed laboratory molecular work (RNA extraction and electrophoresis); G.W. analysed the data; G.W. led the writing of the manuscript. All authors contributed critically to the drafts and gave final approval for publication.

## Funding

Guillaume Wos was supported by the National Science Centre, Poland (grant 2023/51/D/NZ8/01449) and by the Institute of Nature Conservation, Polish Academy of Sciences.

## Ethics Statement

The present study complies with applicable laws on sampling from natural populations and animal experimentation (ARRIVE guidelines).

## Conflicts of Interest

The authors declare no conflicts of interest.

## Supporting information


**Figure S1:** (A) Principal component analysis of seven environmental parameters measured on the eight ponds: Salinity and pH (CPC‐105, Elmetron, Poland), percentage of cropland (cropland; proxy of agricultural pressure) and of forested habitat (forested) (Quantum‐GIS), water temperature for the month April (T_April), May (T_May) and June (T_June) corresponding to the main larval growth period in nature (dataloggers HOBO UA‐001‐64, Onset placed at ~30 cm depth) (Sniegula and Wos 2026). Ponds are coloured based on the percentage of impervious surface and abbreviations are in Table S1. (B) Weekly temperature measured with dataloggers between 2021 and 2025. For some ponds, temperatures were recorded over several years, in that case, we took the average across all years. Each colour represents one pond, the percentage of impervious surface is indicated in parentheses.
**Figure S2:** Dendrogram showing the sample clustering across the 144 individuals sequenced.
**Figure S3:** Selection of soft thresholding power. The scale independence shows the soft thresholding power as a function of signed *R* ^2^ correlation (red line corresponds to *R* ^2^ = 0.8). The mean connectivity represents the average number of connections per gene (red line is set at 50). A soft thresholding power of 4 was retained for the analysis.
**Figure S4:** Dendrogram and the corresponding modules identified by the WGCNA depicted by the different colours.
**Figure S5:** Illustrative examples of a gene with (A) a decrease in expression at 25°C and 28°C compared with 20°C and (B) with an expression going in different directions at 25°C and 28°C compared with 20°C.
**Figure S6:** Illustrative examples of genes showing a significant interaction urbanization × temperature. Only annotated genes with a “Gene name” are shown (Table S3). Colours indicate the temperature treatment: 22°C (blue), 25°C (green) and 28°C (red). Each circle depicts one individual. Grey areas represent standard error.
**Figure S7:** Enriched GO terms and REVIGO clustering of the genes from the brown module identified in the WGCNA.
**Figure S8:** Enriched GO terms and REVIGO clustering of the genes from the grey module identified in the WGCNA.
**Table S1:** Information on individuals used for the gene expression analysis, GPS coordinates and percentage of impervious surface (urbanization level) of the ponds sampled and information on sequencing data.
**Table S2:** Module preservation analysis showing the Z summary scores and median rank for each module. Module preservation analysis was performed on the original dataset used as a reference set and the target set was estimated using permutations (N permutations = 200). Z scores ≤ 2 indicate no module preservation, 2 ≤ Z scores ≤ 10 indicate moderate preservation and Z scores ≥ 10 indicate strong preservation. Median rank lists the different modules from the most preserved (value of 1) to the least preserved (value of 11); the module turquoise and magenta have the same median value and the same rank.
**Table S3:** Lists of genes differentially expressed in response to (A) urbanization, (B) the interaction urbanization × temperature and (C) temperature. For (A) and (B), the GO term enrichment analysis is shown below the list of genes. For (C), the GO term enrichment analysis (or functional categorization) was performed separately on the genes showing (D) a consistent decrease in expression at 25°C and 28°C compared with 22°C (same negative coefficients), (E) a consistent increase in expression (same positive coefficients) and (F) different directions in 25°C and 28°C compared with 22°C (different coefficients).

## Data Availability

Raw sequence data are deposited in the SRA (Bioproject PRJNA1453760). Table S3 is available in Figshare at https://doi.org/10.6084/m9.figshare.33069302.v3.

## References

[mec70511-bib-0001] Alberti, M. , J. Marzluff , and V. M. Hunt . 2017. “Urban Driven Phenotypic Changes: Empirical Observations and Theoretical Implications for Eco‐Evolutionary Feedback.” Philosophical Transactions of the Royal Society, B: Biological Sciences 372, no. 1712: 20160029. 10.1098/rstb.2016.0029 PMC5182425 27920374

[mec70511-bib-0002] Angilletta, M. J., Jr. , T. D. Steury , and M. W. Sears . 2004. “Temperature, Growth Rate, and Body Size in Ectotherms: Fitting Pieces of a Life‐History Puzzle.” Integrative and Comparative Biology 44, no. 6: 498–509. 10.1093/icb/44.6.498. 21676736

[mec70511-bib-0003] Angilletta, M. J. , R. S. Wilson , A. C. Niehaus , M. W. Sears , C. A. Navas , and P. L. Ribeiro . 2007. “Urban Physiology: City Ants Possess High Heat Tolerance.” PLoS One 2, no. 2: e258. 17327918 10.1371/journal.pone.0000258 PMC1797824

[mec70511-bib-0004] Arakane, Y. , and S. Muthukrishnan . 2010. “Insect Chitinase and Chitinase‐Like Proteins.” Cellular and Molecular Life Sciences 67, no. 2: 201–216. 10.1007/s00018-009-0161-9. 19816755 PMC11115512

[mec70511-bib-0005] Ashburner, M. , C. A. Ball , J. A. Blake , et al. 2000. “Gene Ontology: Tool for the Unification of Biology.” Nature Genetics 25, no. 1: 25–29. 10802651 10.1038/75556 PMC3037419

[mec70511-bib-0006] Babik, W. , K. Dudek , M. Marszałek , G. Palomar , B. Antunes , and S. Sniegula . 2023. “The Genomic Response to Urbanization in the Damselfly *Ischnura elegans* .” Evolutionary Applications 16, no. 11: 1805–1818. 10.1111/eva.13603. 38029064 PMC10681423

[mec70511-bib-0007] Brans, K. I. , M. Jansen , J. Vanoverbeke , N. Tüzün , R. Stoks , and L. De Meester . 2017. “The Heat Is on: Genetic Adaptation to Urbanization Mediated by Thermal Tolerance and Body Size.” Global Change Biology 23, no. 12: 5218–5227. 10.1111/gcb.13784. 28614592

[mec70511-bib-0008] Brans, K. I. , R. Stoks , and L. De Meester . 2018. “Urbanization Drives Genetic Differentiation in Physiology and Structures the Evolution of Pace‐Of‐Life Syndromes in the Water Flea *Daphnia magna* .” Proceedings of the Royal Society B: Biological Sciences 285, no. 1883: 20180169. 10.1098/rspb.2018.0169. PMC6083254 30051844

[mec70511-bib-0009] Conrad, K. , K. Willson , I. Harvey , C. Thomas , and T. Sherratt . 1999. “Dispersal Characteristics of Seven Odonate Species in an Agricultural Landscape.” Ecography 22, no. 5: 524–531.

[mec70511-bib-0010] Corbet, P. S. 1999. Dragonflies: Behaviour and Ecology of Odonata. Harley books.

[mec70511-bib-0011] Corbet, P. S. , F. Suhling , and D. Soendgerath . 2006. “Voltinism of Odonata: A Review.” International Journal of Odonatology 9, no. 1: 1–44.

[mec70511-bib-0012] Damiani, G. , M. Sebastiano , G. Dell'Omo , and D. Costantini . 2024. “Blood Transcriptome Analysis of Common Kestrel Nestlings Living in Urban and Non‐Urban Environments.” Science of the Total Environment 928: 172585. 10.1016/j.scitotenv.2024.172585. 38641099

[mec70511-bib-0013] Diamond, S. E. , H. Cayton , T. Wepprich , et al. 2014. “Unexpected Phenological Responses of Butterflies to the Interaction of Urbanization and Geographic Temperature.” Ecology 95, no. 9: 2613–2621.

[mec70511-bib-0014] Dijkstra, K.‐D. , and A. Schröter . 2020. Field Guide to the Dragonflies of Britain and Europe. Bloomsbury Publishing.

[mec70511-bib-0015] Dingle, H. 1991. “Maternal Effects in Insect Life Histories.” Annual Review of Entomology 36, no. 5: 1–34.

[mec70511-bib-0016] EEA . 2020. “European Environment Agency, Copernicus Land Monitoring Service.” https://land.copernicus.eu/pan‐european.

[mec70511-bib-0017] Eggenberger, H. , D. Frey , L. Pellissier , J. Ghazoul , S. Fontana , and M. Moretti . 2019. “Urban Bumblebees Are Smaller and More Phenotypically Diverse Than Their Rural Counterparts.” Journal of Animal Ecology 88, no. 10: 1522–1533. 10.1111/1365-2656.13051. 31233621

[mec70511-bib-0018] Engsontia, P. , U. Sangket , W. Chotigeat , and C. Satasook . 2014. “Molecular Evolution of the Odorant and Gustatory Receptor Genes in Lepidopteran Insects: Implications for Their Adaptation and Speciation.” Journal of Molecular Evolution 79, no. 1: 21–39. 25038840 10.1007/s00239-014-9633-0

[mec70511-bib-0019] Fangue, N. A. , M. Hofmeister , and P. M. Schulte . 2006. “Intraspecific Variation in Thermal Tolerance and Heat Shock Protein Gene Expression in Common Killifish, *Fundulus heteroclitus* .” Journal of Experimental Biology 209, no. 15: 2859–2872. 10.1242/jeb.02260. 16857869

[mec70511-bib-0020] Flenner, I. , and G. Sahlén . 2008. “Dragonfly Community Re‐Organisation in Boreal Forest Lakes: Rapid Species Turnover Driven by Climate Change?” Insect Conservation and Diversity 1, no. 3: 169–179. 10.1111/j.1752-4598.2008.00020.x.

[mec70511-bib-0021] Gall, M. L. , A. Chaput‐Bardy , and A. Husté . 2017. “Context‐Dependent Local Movements of the Blue‐Tailed Damselfly, *Ischnura elegans*: Effects of Pond Characteristics and the Landscape Matrix.” Journal of Insect Conservation 21, no. 2: 243–256. 10.1007/s10841-017-9971-5.

[mec70511-bib-0022] González‐Rete, B. , P. M. Salazar‐Schettino , M. I. Bucio‐Torres , A. Córdoba‐Aguilar , and M. Cabrera‐Bravo . 2019. “Activity of the Prophenoloxidase System and Survival of Triatomines Infected With Different *Trypanosoma cruzi* Strains Under Different Temperatures: Understanding Chagas Disease in the Face of Climate Change.” Parasites & Vectors 12, no. 1: 219. 10.1186/s13071-019-3477-9. 31068226 PMC6507061

[mec70511-bib-0023] González‐Tokman, D. , A. Córdoba‐Aguilar , W. Dáttilo , A. Lira‐Noriega , R. A. Sánchez‐Guillén , and F. Villalobos . 2020. “Insect Responses to Heat: Physiological Mechanisms, Evolution and Ecological Implications in a Warming World.” Biological Reviews 95, no. 3: 802–821. 10.1111/brv.12588. 32035015

[mec70511-bib-0024] Grimm, N. B. , S. H. Faeth , N. E. Golubiewski , et al. 2008. “Global Change and the Ecology of Cities.” Science 319, no. 5864: 756–760. 10.1126/science.1150195. 18258902

[mec70511-bib-0025] Gupta, A. , and S. Nair . 2025. “Epigenetic Processes in Insect Adaptation to Environmental Stress.” Current Opinion in Insect Science 67: 101294. 10.1016/j.cois.2024.101294. 39521342

[mec70511-bib-0026] Harris, S. E. , J. Munshi‐South , C. Obergfell , and R. O'Neill . 2013. “Signatures of Rapid Evolution in Urban and Rural Transcriptomes of White‐Footed Mice ( *Peromyscus leucopus* ) in the New York Metropolitan Area.” PLoS One 8, no. 8: e74938. 10.1371/journal.pone.0074938. 24015321 PMC3756007

[mec70511-bib-0027] Isaksson, C. 2015. “Urbanization, Oxidative Stress and Inflammation: A Question of Evolving, Acclimatizing or Coping With Urban Environmental Stress.” Functional Ecology 29, no. 7: 913–923. 10.1111/1365-2435.12477.

[mec70511-bib-0028] Kelley, J. L. , L. Chapuis , W. I. L. Davies , and S. P. Collin . 2018. “Sensory System Responses to Human‐Induced Environmental Change.” Frontiers in Ecology and Evolution 6: 95. 10.3389/fevo.2018.00095.

[mec70511-bib-0029] Khimoun, A. , C. Doums , M. Molet , et al. 2020. “Urbanization Without Isolation: The Absence of Genetic Structure Among Cities and Forests in the Tiny Acorn Ant *Temnothorax nylanderi* .” Biology Letters 16, no. 1: 20190741. 10.1098/rsbl.2019.0741. 31992150 PMC7013475

[mec70511-bib-0030] Kim, D. , J. M. Paggi , C. Park , C. Bennett , and S. L. Salzberg . 2019. “Graph‐Based Genome Alignment and Genotyping With HISAT2 and HISAT‐Genotype.” Nature Biotechnology 37, no. 8: 907–915. 10.1038/s41587-019-0201-4 PMC7605509 31375807

[mec70511-bib-0031] Konorov, E. A. , M. A. Nikitin , K. V. Mikhailov , et al. 2017. “Genomic Exaptation Enables *Lasius niger* Adaptation to Urban Environments.” BMC Evolutionary Biology 17, no. 1: 39. 10.1186/s12862-016-0867-x. 28251870 PMC5333191

[mec70511-bib-0032] Langfelder, P. , and S. Horvath . 2008. “WGCNA: An R Package for Weighted Correlation Network Analysis.” BMC Bioinformatics 9, no. 1: 1–13. 19114008 10.1186/1471-2105-9-559 PMC2631488

[mec70511-bib-0033] Lewis, M. , K. Goldmann , E. Sciacca , C. Cubuk , and A. Surace . 2021. “glmmSeq: General Linear Mixed Models for Gene‐Level Differential Expression.” R Package Version 0.5.4.

[mec70511-bib-0034] Liao, Y. , G. K. Smyth , and W. Shi . 2019. “The R Package Rsubread Is Easier, Faster, Cheaper and Better for Alignment and Quantification of RNA Sequencing Reads.” Nucleic Acids Research 47, no. 8: e47. 30783653 10.1093/nar/gkz114 PMC6486549

[mec70511-bib-0035] Maere, S. , K. Heymans , and M. Kuiper . 2005. “BiNGO: A Cytoscape Plugin to Assess Overrepresentation of Gene Ontology Categories in Biological Networks.” Bioinformatics (Oxford, England) 21, no. 16: 3448–3449. 10.1093/bioinformatics/bti551. 15972284

[mec70511-bib-0036] Magnusson, A. , H. Skaug , A. Nielsen , et al. 2017. “Package ‘glmmTMB.’ R Package Version 0.2.0.”

[mec70511-bib-0037] Martin, M. 2011. “Cutadapt Removes Adapter Sequences From High‐Throughput Sequencing Reads.” EMBnet.journal 17, no. 1: 10–12.

[mec70511-bib-0038] Masson‐Delmotte, V. , P. Zhai , A. Pirani , et al. 2021. Climate Change 2021: The Physical Science Basis. Contribution of Working Group I to the Sixth Assessment Report of the Intergovernmental Panel on Climate Change. Cambridge University Press.

[mec70511-bib-0039] McNew, S. M. , D. Beck , I. Sadler‐Riggleman , et al. 2017. “Epigenetic Variation Between Urban and Rural Populations of Darwin's Finches.” BMC Evolutionary Biology 17, no. 1: 183. 10.1186/s12862-017-1025-9. 28835203 PMC5569522

[mec70511-bib-0040] Meillère, A. , F. Brischoux , C. Parenteau , and F. Angelier . 2015. “Influence of Urbanization on Body Size, Condition, and Physiology in an Urban Exploiter: A Multi‐Component Approach.” PLoS One 10, no. 8: e0135685. 26270531 10.1371/journal.pone.0135685 PMC4535910

[mec70511-bib-0041] Merckx, T. , A. Kaiser , and H. Van Dyck . 2018. “Increased Body Size Along Urbanization Gradients at Both Community and Intraspecific Level in Macro‐Moths.” Global Change Biology 24, no. 8: 3837–3848. 10.1111/gcb.14151. 29791767

[mec70511-bib-0042] Miles, L. S. , L. R. Rivkin , M. T. J. Johnson , J. Munshi‐South , and B. C. Verrelli . 2019. “Gene Flow and Genetic Drift in Urban Environments.” Molecular Ecology 28, no. 18: 4138–4151. 10.1111/mec.15221. 31482608

[mec70511-bib-0043] Morse, C. C. , A. D. Huryn , and C. Cronan . 2003. “Impervious Surface Area as a Predictor of the Effects of Urbanization on Stream Insect Communities in Maine, U.S.A.” Environmental Monitoring and Assessment 89, no. 1: 95–127. 10.1023/A:1025821622411. 14609276

[mec70511-bib-0044] Mueller, J. C. , J. Partecke , B. J. Hatchwell , K. J. Gaston , and K. L. Evans . 2013. “Candidate Gene Polymorphisms for Behavioural Adaptations During Urbanization in Blackbirds.” Molecular Ecology 22, no. 13: 3629–3637. 10.1111/mec.12288. 23495914

[mec70511-bib-0045] Norling, U. 2021. “Growth, Winter Preparations and Timing of Emergence in Temperate Zone Odonata: Control by a Succession of Larval Response Patterns.” International Journal of Odonatology 24: 1–36.

[mec70511-bib-0046] Palomar, G. , G. Wos , R. Stoks , and S. Sniegula . 2023. “Latitude‐Specific Urbanization Effects on Life History Traits in the Damselfly *Ischnura elegans* .” Evolutionary Applications 16, no. 8: 1503–1515. 10.1111/eva.13583. 37622092 PMC10445092

[mec70511-bib-0047] QGIS Development Team . 2017. “QGIS Geographic Information System.” Open Source Geospatial Foundation Project.

[mec70511-bib-0048] R Core Team . 2024. R: A Language and Environment for Statistical Computing. R Foundation for Statistical Computing. https://www.gbif.org/tool/81287/r‐a‐language‐and‐environment‐for‐statistical‐computing.

[mec70511-bib-0049] Rho, M. S. , and K. P. Lee . 2017. “Temperature‐Driven Plasticity in Nutrient Use and Preference in an Ectotherm.” Oecologia 185, no. 3: 401–413. 10.1007/s00442-017-3959-4. 28932986

[mec70511-bib-0050] Robinson, M. D. , D. J. McCarthy , and G. K. Smyth . 2010. “edgeR: A Bioconductor Package for Differential Expression Analysis of Digital Gene Expression Data.” Bioinformatics 26, no. 1: 139–140. 10.1093/bioinformatics/btp616. 19910308 PMC2796818

[mec70511-bib-0051] RStudio Team . 2024. RStudio: Integrated Development for R. RStudio, Inc. http://www.rstudio.com/.

[mec70511-bib-0052] Santangelo, J. S. , L. R. Rivkin , C. Advenard , and K. A. Thompson . 2020. “Multivariate Phenotypic Divergence Along an Urbanization Gradient.” Biology Letters 16, no. 9: 20200511. 10.1098/rsbl.2020.0511. 32991825 PMC7532719

[mec70511-bib-0053] Shannon, P. , A. Markiel , O. Ozier , et al. 2003. “Cytoscape: A Software Environment for Integrated Models of Biomolecular Interaction Networks.” Genome Research 13, no. 11: 2498–2504. 10.1101/gr.1239303. 14597658 PMC403769

[mec70511-bib-0054] Smith, N. M. A. , L. Jacquier , E. Gay , M. Molet , and C. Doums . 2025. “The Transcriptome of the Ant Temnothorax Nylanderi Is Not Affected by Urbanisation but by Rearing Conditions.” Insect Molecular Biology 34, no. 6: 889–899. 10.1111/imb.13011. 40579956

[mec70511-bib-0055] Sniegula, S. , and G. Wos . 2026. “Urbanization Drives Phenotypic Differences but Not Asymmetry in the Damselfly *Ischnura elegans* .” Scientific Reports. 10.1038/s41598-026-57538-7. PMC13526801 42289478

[mec70511-bib-0056] Supek, F. , M. Bošnjak , N. Škunca , and T. Šmuc . 2011. “REVIGO Summarizes and Visualizes Long Lists of Gene Ontology Terms.” PLoS One 6, no. 7: e21800. 10.1371/journal.pone.0021800. 21789182 PMC3138752

[mec70511-bib-0057] Swaegers, J. , K. I. Spanier , and R. Stoks . 2020. “Genetic Compensation Rather Than Genetic Assimilation Drives the Evolution of Plasticity in Response to Mild Warming Across Latitudes in a Damselfly.” Molecular Ecology 29, no. 24: 4823–4834. 10.1111/mec.15676. 33031581

[mec70511-bib-0058] Tam, B. Y. , W. A. Gough , and T. Mohsin . 2015. “The Impact of Urbanization and the Urban Heat Island Effect on Day to Day Temperature Variation.” Urban Climate 12: 1–10. 10.1016/j.uclim.2014.12.004.

[mec70511-bib-0059] The Darwin Tree of Life Project Consortium . 2022. “Sequence Locally, Think Globally: The Darwin Tree of Life Project.” Proceedings of the National Academy of Sciences of the United States of America 119, no. 4: e2115642118. 35042805 10.1073/pnas.2115642118 PMC8797607

[mec70511-bib-0060] Theodorou, P. , R. Radzevičiūtė , B. Kahnt , A. Soro , I. Grosse , and R. J. Paxton . 2018. “Genome‐Wide Single Nucleotide Polymorphism Scan Suggests Adaptation to Urbanization in an Important Pollinator, the Red‐Tailed Bumblebee ( *Bombus lapidarius* L.).” Proceedings of the Royal Society B: Biological Sciences 285, no. 1877: 20172806. 10.1098/rspb.2017.2806. PMC5936727 29669900

[mec70511-bib-0061] Thompson, M. J. , P. Capilla‐Lasheras , D. M. Dominoni , D. Réale , and A. Charmantier . 2022. “Phenotypic Variation in Urban Environments: Mechanisms and Implications.” Trends in Ecology & Evolution 37, no. 2: 171–182. 10.1016/j.tree.2021.09.009. 34690006

[mec70511-bib-0062] Tüzün, N. , L. Op de Beeck , K. I. Brans , L. Janssens , and R. Stoks . 2017. “Microgeographic Differentiation in Thermal Performance Curves Between Rural and Urban Populations of an Aquatic Insect.” Evolutionary Applications 10, no. 10: 1067–1075. 10.1111/eva.12512. 29151861 PMC5680628

[mec70511-bib-0063] Tüzün, N. , and R. Stoks . 2021. “Lower Bioenergetic Costs but Similar Immune Responsiveness Under a Heat Wave in Urban Compared to Rural Damselflies.” Evolutionary Applications 14, no. 1: 24–35. 10.1111/eva.13041. 33519954 PMC7819556

[mec70511-bib-0064] Uniprot Consortium . 2015. “UniProt: A Hub for Protein Information.” Nucleic Acids Research 43, no. D1: D204–D212. 25348405 10.1093/nar/gku989 PMC4384041

[mec70511-bib-0065] van Vliet, J. 2019. “Direct and Indirect Loss of Natural Area From Urban Expansion.” Nature Sustainability 2, no. 8: 755–763. 10.1038/s41893-019-0340-0.

[mec70511-bib-0066] Ward, J. V. , and J. A. Stanford . 1982. “Thermal Responses in the Evolutionary Ecology of Aquatic Insects.” Annual Review of Entomology 27: 97–117. 10.1146/annurev.en.27.010182.000525.

[mec70511-bib-0067] Watson, H. , E. Videvall , M. N. Andersson , and C. Isaksson . 2017. “Transcriptome Analysis of a Wild Bird Reveals Physiological Responses to the Urban Environment.” Scientific Reports 7, no. 1: 44180. 10.1038/srep44180. 28290496 PMC5349542

[mec70511-bib-0068] Watts, P. C. , S. Keat , and D. J. Thompson . 2010. “Patterns of Spatial Genetic Structure and Diversity at the Onset of a Rapid Range Expansion: Colonisation of the UK by the Small Red‐Eyed Damselfly *Erythromma viridulum* .” Biological Invasions 12, no. 11: 3887–3903. 10.1007/s10530-010-9779-7.

[mec70511-bib-0069] Winchell, K. M. , S. C. Campbell‐Staton , J. B. Losos , L. J. Revell , B. C. Verrelli , and A. J. Geneva . 2023. “Genome‐Wide Parallelism Underlies Contemporary Adaptation in Urban Lizards.” Proceedings of the National Academy of Sciences of the United States of America 120, no. 3: e2216789120. 10.1073/pnas.2216789120. 36634133 PMC9934206

[mec70511-bib-0070] Wos, G. , G. Palomar , M. Marszałek , W. Babik , and S. Sniegula . 2023. “The Effect of Temperature and Invasive Alien Predator on Genetic and Phenotypic Variation in the Damselfly *Ischnura elegans*: Cross‐Latitude Comparison.” Frontiers in Zoology 20, no. 1: 13. 10.1186/s12983-023-00494-z. 37032330 PMC10084621

[mec70511-bib-0071] Wos, G. , R. Stoks , and S. Sniegula . 2026. “Seasonal Time Constraints Reduce the Impact of Warming on Life History but Not on Physiology.” Ecological Entomology 51, no. 4: 747–759. 10.1111/een.70087.

[mec70511-bib-0072] Yauk, C. , A. Polyzos , A. Rowan‐Carroll , et al. 2008. “Germ‐Line Mutations, DNA Damage, and Global Hypermethylation in Mice Exposed to Particulate Air Pollution in an Urban/Industrial Location.” Proceedings of the National Academy of Sciences 105, no. 2: 605–610. 10.1073/pnas.0705896105. PMC2206583 18195365

[mec70511-bib-0073] Yeaman, S. , and M. C. Whitlock . 2011. “The Genetic Architecture of Adaptation Under Migration–Selection Balance.” Evolution 65, no. 7: 1897–1911. 10.1111/j.1558-5646.2011.01269.x. 21729046

