# Supplementary material for: The Transcriptomic Response Along an Urbanization Gradient Is Also Temperature‐Dependent in a Freshwater Insect
Source: Mol Ecol. 2026 Aug 17;35(16):e70511. doi: 10.1111/mec.70511 (PMC13480148; doi:10.1111/mec.70511)
Supplement: Supplementary file 1 [file MEC-35-e70511-s001.zip › mec70511-sup-0001-TableS2-S2-FigureS1-S8@MEC_Supplemental_Information.docx]

**Supplemental Information for:**

**The transcriptomic response along an urbanization gradient is also temperature-dependent in a freshwater insect**

Katarzyna Dudek^1^, Guillaume Wos^2,^*

^1^Institute of Environmental Sciences, Jagiellonian University, Gronostajowa 7, 30-387 Kraków, Poland

^2^Institute of Nature Conservation Polish Academy of Sciences, al. Adama Mickiewicza 33, 31-120 Krakow, Poland

* Author for correspondence:

Guillaume Wos

[wos.gui@gmail.com](mailto:wos.gui@gmail.com)

**Table of Contents:**

| **Table S2** | Page 2 |
| --- | --- |
| **Fig. S1** | Page 3 |
| **Fig. S2** | Page 5 |
| **Fig. S3** | Page 6 |
| **Fig. S4** | Page 7 |
| **Fig. S5** | Page 8 |
| **Fig. S6** | Page 9 |
| **Fig. S7** | Page 11 |
| **Fig. S8** | Page 12 |

**Table S2**. Module preservation analysis showing the Z summary scores and median rank for each module. Module preservation analysis was performed on the original dataset used as a reference set and the target set was estimated using permutations (N permutations = 200). Z scores ≤ 2 indicate no module preservation, 2 ≤ Z scores ≤ 10 indicate moderate preservation and Z scores ≥ 10 indicate strong preservation. Median rank lists the different modules from the most preserved (value of 1) to the least preserved (value of 11); the module turquoise and magenta have the same median value and the same rank.

| Module | Z summary | Median rank |
| --- | --- | --- |
| ME turquoise | 26.80101 | 1 |
| ME magenta | 15.02832 | 1 |
| ME red | 28.20475 | 2 |
| ME yellow | 29.17376 | 3 |
| ME pink | 17.90645 | 4 |
| ME greenyellow | 12.76819 | 5 |
| ME purple | 14.0846 | 6 |
| ME brown | 7.73043 | 7 |
| ME grey | 60.95067 | 8 |
| ME green | 29.78593 | 9 |
| ME blue | 8.307924 | 10 |
| ME black | 23.82181 | 11 |

**Fig. S1.** A) Principal component analysis of seven environmental parameters measured on the eight ponds: Salinity and pH (CPC-105, Elmetron, Poland), percentage of cropland (cropland; proxy of agricultural pressure) and of forested habitat (forested) (Quantum-GIS), water temperature for the month April (T_April), May (T_May) and June (T_June) corresponding to the main larval growth period in nature (dataloggers HOBO UA-001-64, Onset placed at ~ 30 cm depth) (Sniegula & Wos, 2026). Ponds are colored based on the percentage of impervious surface and abbreviations are in Table S1. B) Weekly temperature measured with dataloggers between 2021 and 2025. For some ponds, temperatures were recorded over several years, in that case, we took the average across all years. Each color represents one pond, the percentage of impervious surface is indicated in parentheses.

**Fig. S2**. Dendrogram showing the sample clustering across the 144 individuals sequenced.

**Fig. S3**. Selection of soft thresholding power. The scale independence shows the soft thresholding power as a function of signed R^2^ correlation (red line corresponds to R^2^ = 0.8). The mean connectivity represents the average number of connections per gene (red line is set at 50). A soft thresholding power of 4 was retained for the analysis.

**Fig S4**. Dendrogram and the corresponding modules identified by the WGCNA depicted by the different colors.

**Fig. S5**. Illustrative examples of a gene with A) a decrease in expression at 25°C and 28°C compared with 20°C and B) with an expression going in different directions at 25°C and 28°C compared with 20°C.

**Fig. S6**. Illustrative examples of genes showing a significant interaction urbanization × temperature. Only annotated genes with a “Gene name” are shown (Table S3). Colors indicate the temperature treatment: 22°C (blue), 25°C (green) and 28°C (red). Each circle depicts one individual. Grey areas represent standard error.

**Fig. S7**. Enriched GO terms and REVIGO clustering of the genes from the brown module identified in the WGCNA.

**Fig. S8**. Enriched GO terms and REVIGO clustering of the genes from the grey module identified in the WGCNA.
